# Morpho-Phylogenetic Evidence Reveals New Species of Fuscosporellaceae and Savoryellaceae from Freshwater Habitats in Guizhou Province, China

**DOI:** 10.3390/jof8111138

**Published:** 2022-10-28

**Authors:** Hong-Zhi Du, Jing Yang, Ning-Guo Liu, Ratchadawan Cheewangkoon, Jian-Kui Liu

**Affiliations:** 1 School of Life Science and Technology, Center for Informational Biology, University of Electronic Science and Technology of China, Chengdu 611731, China; 2 School of Pharmacy, Guizhou University of Traditional Chinese Medicine, Guiyang 550025, China; 3 Department of Entomology and Plant Pathology, Faculty of Agriculture, Chiang Mai University, Chiang Mai 50200, Thailand; 4 Center of Excellence in Fungal Research, Mae Fah Luang University, Chiang Rai 57100, Thailand

**Keywords:** 3 new taxa, asexual fungi, multi-gene, wood-inhabiting fungi, sordariomycetes, taxonomy

## Abstract

During a survey of freshwater fungi in Guizhou Province, China, six hyphomycetous collections were founded on decaying wood from freshwater habitats. These taxa were characterized and identified based on morphology, phylogeny, and culture characteristics. Phylogenetic analysis of combined LSU, SSU, ITS, *RPB2* and *TEF1α* sequence data indicated that our six isolates formed three distinct lineages and were distributed within Fuscosporellaceae and Savoryellaceae. They can be organized as three new species: *Fuscosporella guizhouensis*, *Mucispora* *aquatica* and *Neoascotaiwania* *guizhouensis*. *Fuscosporella guizhouensis* and *Neoascotaiwania guizhouensis* have sporodochial conidiomata, micronematous conidiophores and dark brown conidia. The former possesses irregularly ellipsoidal conidia with apical appendages, while the latter has fusiform to obovoid conidia. *Mucispora aquatica* is characterized by macronematous conidiophores, elongating percurrently and dark brown, narrowly obovoid conidia. The detailed, illustrated descriptions and notes for each new taxon are provided, and the species of *Fuscosporella* is reported for the first time in China.

## 1. Introduction

Freshwater fungi were defined as ‘‘fungi that the whole or part of their life cycle rely on freshwater’’ [1]. They are a diverse and heterogeneous group comprising different species and play an essential role in the organic carbon cycle of aquatic ecosystems [2,3]. Freshwater habitats include lentic and lotic water [1,2] and artificial habitats [4,5]. Calabon et al. [6] recently gave a comprehensive review of the freshwater fungal numbers and listed 3870 freshwater fungal species. Two hundred and ninety-eight novel species have been described in China and Thailand from 2015 to 2020 [6]. It is worth noting that the application of molecular techniques coupled with traditional morphology has significantly improved fungal identification and classification, especially the phylogenetic relationships of freshwater taxa.

The monotypic order Fuscosporellales was introduced by Yang et al. [7], with Fuscosporellaceae as the type family, based on phylogenetic analyses, and six genera were assigned, viz. *Bactrodesmiastrum*, *Fuscosporella* (type genus), *Mucispora*, *Parafuscosporella*, *Plagiascoma,* and *Pseudoascotaiwania* [7]. *Plagiascoma* and *Pseudoascotaiwania* are known for their sexual morphs, which have immersed to semi-immersed, dark brown to black ascomata, unitunicate, cylindrical to cylindric-fusiform, stipitate, 8-spored asci with a non-amyloid apical ring, and uniseriate, hyaline or light brown, fusiform, septate ascospores [7,8]. Asexual genera *Bactrodesmiastrum*, *Fuscosporella,* and *Parafuscosporella* share the features of having sporodochial conidiomata, semi-macronematous to macronematous, hyaline to brown, smooth-walled conidiophores, monoblastic, integrated, hyaline to dark brown conidiogenous cells and ellipsoidal, obovoid to pyriform, brown to dark brown, septate conidia [7,9,10]. In comparison, *Mucispora* is distinct in having macronematous, mononematous, solitary, erect, brown conidiophores, usually elongating percurrently, and ellipsoidal to obovoid conidia, sometimes with a hyaline mucilaginous sheath [7,11].

Boonyuen et al. [12] established Savoryellales to accommodate *Ascotaiwania*, *Canalisporium* (=*Ascothailandia*), and *Savoryella* based on multi-gene analyses (LSU, SSU, 5.8S rDNA, *RPB1*, *RPB2* and *TEF1α*). They are distributed in freshwater, brackish, marine and terrestrial habitats, and Savoryellaceae was later formally introduced by Jaklitsch and Réblová [13]. Subsequently, Hernández-Restrepo et al. [14] introduced a bactrodesmium-like genus *Neoascotaiwania*, and Luo et al. [15] added a monotypic and monodictys-like genus *Dematiosporium* in Savoryellaceae. Réblová et al. [16] assessed the systematic placement of several *Bactrodesmium* species within Savoryellaceae. Sexual morphs of Savoryellales have non-stromatic, immersed, semi-immersed to superficial, dark, coriaceous ascomata, clavate to cylindrical unitunicate asci with a non-amyloid apical ring, ellipsoid to fusiform, transversely septate ascospores with hyaline polar cells and brown middle cells. Asexual morphs in Savoryellales are characterized by semi-macronematous conidiophores, monoblastic conidiogenous cells and transversely septate or dictyoseptate conidia [4,8,12,17,18,19]. Fuscosporellales and Savoryellales were initially placed in Hypocreomycetidae (Sordariomycetes) [7,12], whereafter, based on the phylogenetic and molecular clock analyses, they were referred to as a new subclass of Savoryellomycetidae (Sordariomycetes) along with Conioscyphales and Pleurotheciales by Hongsanan et al. [20].

Six isolates were obtained from submerged decaying wood during the survey of freshwater fungi in Guizhou Province, China. This study aims to describe these new findings and contribute to fungal diversity in China. Morphological comparison coupled with multi-gene phylogeny was carried out to determine the classification of these new collections. As a result, three new species are introduced, and the establishment of these new taxa is justified by morphology and phylogenetic evidence.

## 2. Materials and Methods

### 2.1. Collection and Examination of Specimens

Specimens of submerged decaying wood were collected from a freshwater stream in Guizhou Province, China, in February 2021. Samples were brought to the laboratory in plastic bags and incubated in plastic boxes lined with moistened tissue paper at room temperature for one week. Morphological observations were made using a Motic SMZ (Stereoscopic Zoom Microscope) 168 Series dissecting microscope (Motic, Xiamen, China) for fungal structures on a natural substrate. The fruiting bodies were collected using a syringe needle and transferred to a drop of tap water on a clean slide. The features were examined and photographed using a Nikon ECLIPSE Ni-U compound microscope fitted with a Nikon DS-Ri2 digital camera. Measurements were made with the Tarosoft Image Frame Work v. 0.9.7 software following the procedures outlined by Liu et al. [21], and images used for photo plates were processed with Adobe Photoshop CC 2018 software (Adobe Systems, San Jose, CA, USA). Single-spore isolations were made on potato dextrose agar (PDA) or water agar (WA) and later transferred onto new PDA plates following the methods described in Senanayake et al. [22]. Incubation and cultural growth were observed at 25 °C.

Herbarium specimens were deposited in the Herbarium of Cryptogams, Kunming Institute of Botany Academia Sinica (HKAS), Kunming, China, and Herbarium, University of Electronic Science and Technology (HUEST), Chengdu, China. The pure cultures obtained in this study were deposited in the China General Microbiological Culture Collection Center (CGMCC) in Beijing, China, and the University of Electronic Science and Technology Culture Collection (UESTCC), Chengdu, China. The new taxa were registered in MycoBank (2022).

### 2.2. DNA Extraction, PCR Amplification and Sequencing

Isolates grew in PDA medium at 25 °C for one month. Fungal mycelia were scraped off and transferred to 1.5 mL microcentrifuge tubes using a sterilized lancet for genomic DNA extraction. A Tsingke Fungus Genomic DNA Extraction Kit (Tsingke Biotech, Shanghai, China) was used to extract DNA following the manufacturer’s instructions. Five gene regions were amplified by Polymerase Chain Reaction (PCR). The nuclear large subunit rDNA (28S, LSU), nuclear small subunit rDNA (18S, SSU), internal transcribed spacer (ITS), RNA polymerase second-largest subunit (*RPB2*) and translation elongation factor 1-alpha (*TEF1α*) were selected for the study. The primers used were LR0R/LR5 for LSU [23], NS1/NS4 for SSU [24], ITS5/ITS4 for ITS [24], fRPB2-5F and fRPB2-7cR for *RPB2* [25] and TEF1-983F/TEF1-2218R for *TEF1α* [26]. The amplifications were performed in a 25 μL reaction volume containing 9.5 μL of ddH_2_O, 12.5 μL of 2 × Taq PCR Master Mix with blue dye (Sangon Biotech, Shanghai, China), 1 μL of DNA template and 1 μL of each primer. The amplification condition for ITS, LSU, SSU and *TEF1α* consisted of initial denaturation at 94 °C for 3 min, followed by 40 cycles of 45 s at 94 °C, 50 s at 55 °C and 1 min at 72 °C, and a final extension period of 10 min at 72 °C. The amplification condition for the *RPB2* gene consisted of initial denaturation at 95 °C for 5 min; followed by 37 cycles of 15 s at 95 °C, 50 s at 56 °C and 2 min at 72 °C, and a final extension period of 10 min at 72 °C. The PCR product purification and sequencing were performed at Beijing Tsingke Biotechnology (Chengdu) Co., Ltd., Chengdu, China.

### 2.3. Phylogenetic Analyses

In this study, the taxa included in the phylogenetic analyses were selected and obtained from previous studies and GenBank (Table 1), with a total of 50 taxa, including four orders, namely, Conioscyphales, Fuscosporellales, Pleurotheciales and Savoryellales. *Tolypocladium capitatum* (OSC 110991) and *T. japonicum* (OSC 71233) (Hypocreales) were selected as outgroup taxa. Single-gene alignments were made in MAFFT v. 7 (http://mafft.cbrc.jp/alignment/server/ (accessed on 7 May 2022)) [27] and checked visually using AliView [28]. The alignments were trimmed using trimAl v 1.2 [29] with minimal coverage (-cons) = 0.8 and gap threshold (-gt) = 0.6. Five single-gene alignments were combined using SequenceMatrix 1.7.8 [30]. Maximum likelihood (ML), Bayesian inference (BI) and maximum parsimony (MP) analyses were employed to assess phylogenetic relationships as detailed in Dissanayake et al. [31].

ML analyses were performed with RAxML-HPC v.8 on XSEDE (8.2.12) [32,33] through the CIPRES Science Gateway V. 3.3 (https://www.phylo.org/portal2/login!input.action (accessed on 18 May 2022)) [34]. The tree search included 1000 non-parametric bootstrap replicates; the best scoring tree was selected among suboptimal trees from each run by comparing likelihood scores under the GTRGAMMA substitution model. The resulting replicates were plotted on to the best scoring tree obtained previously. ML bootstrap values equal to or greater than 75% were marked near each node.

BI was performed in MrBayes 3.2.6 [35]. The program MrModeltest 2 v. 2.3 [36] was used to determine the best nucleotide substitution model for each data partition. The GTR + I+G substitution model was decided for all LSU, SSU, ITS, *RPB2* and *TEF1α* genes. Posterior probabilities (PP) [37] were determined by Markov chain Monte Carlo sampling (MCMC). Six simultaneous Markov chains were run for 10 million generations, and trees were sampled every 1000th generation. The first 25% of the saved trees, representing the burn-in phase of the analysis, were discarded. The remaining trees were used for calculating posterior probabilities in the majority rule consensus tree [38]. PP values equal to or greater than 0.95 were marked near each node.

MP analyses with the heuristic search were performed in PAUP v. 4.0 b10 [39]. The gaps in the alignment were treated as missing characters, and all characters were unordered. Maxtrees were unlimited, branches of zero length were collapsed, and all multiple, equally parsimonious trees were saved. Clade stability was assessed using a bootstrap (BT) analysis with 1000 replicates, each with 10 replicates of random stepwise addition of taxa [40]. MP bootstrap values equal to or greater than 75% were marked near each node.

Phylogenetic trees were printed with Fig Tree v. 1.4.4 (http://tree.bio.ed.ac.uk/software/figtree/ accessed on 18 July 2022)) and the layout was created in Adobe Illustrator CS6 software (Adobe Systems, Inc., San Jose, CA, USA). The sequences generated in this study were deposited in GenBank (Table 1).

**Table 1:** Taxa used in the phylogenetic analyses and the corresponding GenBank accession numbers.

**Taxon**	**Source**	**GenBank Accession Number**					**References**
		**LSU**	**SSU**	**ITS**	***RPB2***	***TEF1α***	
*Ascotaiwania latericolla*	ICMP 22739 ^T^	MN699407	–	MN699390	MN704312	–	[16]
*Ascotaiwania lignicola*	NIL 00006	HQ446365	HQ446285	HQ446342	–	HQ446308	[12]
*Bactrodesmiastrum obovatum*	FMR 6482 ^T^	FR870266	–	FR870264	–	–	[41]
*Bactrodesmiastrum pyriforme*	FMR 10747 ^T^	FR870265	–	FR870263	–	–	[41]
*Bactrodesmiastrum pyriforme*	FMR 11931	HE646637	–	HE646636	–	–	[41]
*Bactrodesmiastrum monilioides*	FMR 10756	KF771879	–	KF771878	–	–	[10]
*Bactrodesmium leptopus*	CBS 144542	MN699423	MN699374	MN699388	MN704297	MN704321	[16]
*Bactrodesmium obovatum*	CBS 144407	MN699426	MN699377	MN699397	MN704299	MN704324	[16]
*Canalisporium elegans*	SS 00895	GQ390271	GQ390256	–	HQ446425	HQ446311	[12]
*Canalisporium caribense*	SS 03683	GQ390269	GQ390254	–	–	–	[12]
*Canalisporium grenadoidia*	BCC 20507 ^T^	GQ390267	GQ390252	GQ390282	HQ446420	HQ446309	[12]
*Conioscypha hoehnelii*	FMR 11592 ^T^	KY853497	HF937348	KY853437	–	–	[14]
*Conioscypha japonica*	CBS 387.84 ^T^	AY484514	JQ437438	–	JQ429259	–	[42,43]
*Conioscypha lignicola*	CBS 335.93 ^T^	AY484513	JQ437439	–	JQ429260	–	[42,43]
*Conioscypha varia*	CBS 113653	AY484512	AY484511	–	JQ429261	–	[42,43]
*Dematiosporium aquaticum*	MFLU 18-1641	MK835855	–	–	MN194029	MN200286	[15]
*Fuscosporella aquatica*	MFLUCC 16-0859	MG388209	–	MG388212	–	–	[44]
***Fuscosporella guizhouensis***	**CGMCC 3.20884** ^T^	**OP376725**	**OP376721**	**OP376715**	**OP367755**	**OP367761**	**This study**
***Fuscosporella guizhouensis***	**UESTCC 22.0017**	**OP376729**	**OP376720**	**OP376727**	**OP367756**	**OP367762**	**This study**
*Fuscosporella pyriformis*	MFLUCC 16-0570 ^T^	KX550896	KX550900	MG388217	KX576872	–	[7]
***Mucispora aquatica***	**CGMCC 3.20882** ^T^	**OP376717**	**OP376726**	**OP376713**	**OP367752**	**OP367757**	**This study**
***Mucispora aquatica***	**UESTCC 22.0018**	**OP376716**	**OP376718**	**OP376712**	**–**	**OP367758**	**This study**
*Mucispora infundibulata*	MFLUCC 16-0866 ^T^	MH457139	MH457171	MH457174	–	–	[11]
*Mucispora obscuriseptata*	MFLUCC 15-0618 ^T^	KX550892	KX550897	MG388218	KX576870	–	[7]
*Mucispora phangngaensis*	MFLUCC 16-0865	MG388210	–	MG388213	–	–	[44]
*Neoascotaiwania fusiformis*	MFLUCC 15-0621 ^T^	KX550893	–	MG388215	KX576871	–	[7]
*Neoascotaiwania fusiformis*	MFLUCC 15-0625	KX550894	KX550898	MG388216	–	–	[7]
***Neoascotaiwania guizhouensis***	**CGMCC 3.20883** ^T^	**OP376731**	**OP376719**	**OP376728**	**OP367753**	**OP367759**	**This study**
***Neoascotaiwania guizhouensis***	**UESTCC 22.0019**	**OP718560**	**–**	**OP376730**	**OP367754**	**OP367760**	**This study**
*Neoascotaiwania limnetica*	CBS 126576	KY853513	KT278689	KY853452	MN704308	MN704331	[8,14,16]
*Neoascotaiwania limnetica*	CBS 126792	KY853514	KT278690	KY853453	MN704309	MN704332	[8,14,16]
*Neoascotaiwania terrestris*	CBS 144402	MN699434	MN699386	MN699405	MN704310	MN704333	[16]
*Neoascotaiwania terrestris*	CBS 142291 ^T^	KY853515	KY853547	KY853454	–	–	[14,16]
*Parafuscosporella* *moniliformis*	MFLUCC 15-0626 ^T^	KX550895	KX550899	MG388219	–	–	[7]
*Parafuscosporella mucosa*	MFLUCC 16-0571 ^T^	MG388211	–	MG388214	–	–	[7]
*Parafuscosporella pyriformis*	KUMCC 19-0008	MN512340	–	MN513031	–	–	[45]
*Parafuscosporella garethii*	FF00725.01 ^T^	KX958430	KX958429	–	KX958432	–	[46]
*Parafuscosporella aquatica*	KUMCC 19-0211 ^T^	MN512343	–	MN513034	–	–	[45]
*Phaeoisaria aquatica*	MFLUCC 16-1298 ^T^	MF399254	–	MF399237	MF401406	–	[47]
*Phaeoisaria fasciculata*	CBS 127885 ^T^	KT278705	KT278693	KT278719	KT278741	–	[8]
*Plagiascoma frondosum*	CBS 139031 ^T^	KT278713	KT278701	–	KT278749	–	[8]
*Pleurotheciella erumpens*	CBS 142447 ^T^	MN699435	MN699387	MN699406	MN704311	MN704334	[8]
*Pleurotheciella guttulata*	KUMCC 15-0296 ^T^	MF399257	MF399223	MF399240	MF401409	–	[47]
*Pleurothecium aquaticum*	MFLUCC 17-1331 ^T^	MF399263	–	MF399245	–	–	[47]
*Pleurothecium floriforme*	MFLUCC 15-1163 ^T^	KY697277	KY697279	KY697281	–	–	[48]
*Pseudoascotaiwania persoonii*	A57 14C ^T^	AY094190	–	–	–	–	[49]
*Savoryella lignicola*	NF 00204	HQ446378	HQ446300	HQ446357	–	HQ446334	[12]
*Savoryella nypae*	MFLUCC 18-1570	MK543210	MK543237	MK543219	–	MK542516	[50]
*Tolypocladium capitatum*	OSC 71233	AY489721	AY489689	–	DQ522421	AY489615	[51,52]
*Tolypocladium japonicum*	OSC 110991	DQ518761	DQ522547	–	DQ522428	DQ522330	[52]

Remarks: The superscript T denotes ex-type isolates. “−” denotes the sequence is unavailable. The newly generated sequences and new species are indicated in bold. **Abbreviations: BCC:** BIOTEC Culture Collection, Bangkok, Thailand; **CBS:** CBS−KNAW Fungal Biodiversity Centre, Utrecht, The Netherlands; **CGMCC:** China General Microbiological Culture Collection Center, Institute of Microbiology, Chinese Academy of Sciences, Beijing, China; **FMR:** Facultat de Medicina i Ciencies de la Salut, Reus, Spain; **ICMP:** International Collection of Microorganisms from Plants, Auckland, New Zealand; **ILLS:** University of Illinois Fungus Collection, Illinois, America; **KUMCC:** Kunming Institute of Botany Culture Collection, Kunming, China; **MFLU:** Mae Fah Luang University Herbarium Collection, Chiang Rai, Thailand; **MFLUCC:** Mae Fah Luang University Culture Collection, Chiang Rai, Thailand; **OSC:** Oregon State University Herbarium, Oregon, America; **UESTCC:** University of Electronic Science and Technology Culture Collection, Chengdu, China; Isolates with the prefix **NF, NIL** and **SS**, **SAT** are from the BIOTEC Culture Collection (BCC).

## 3. Phylogenetic Results

**Figure 1:**
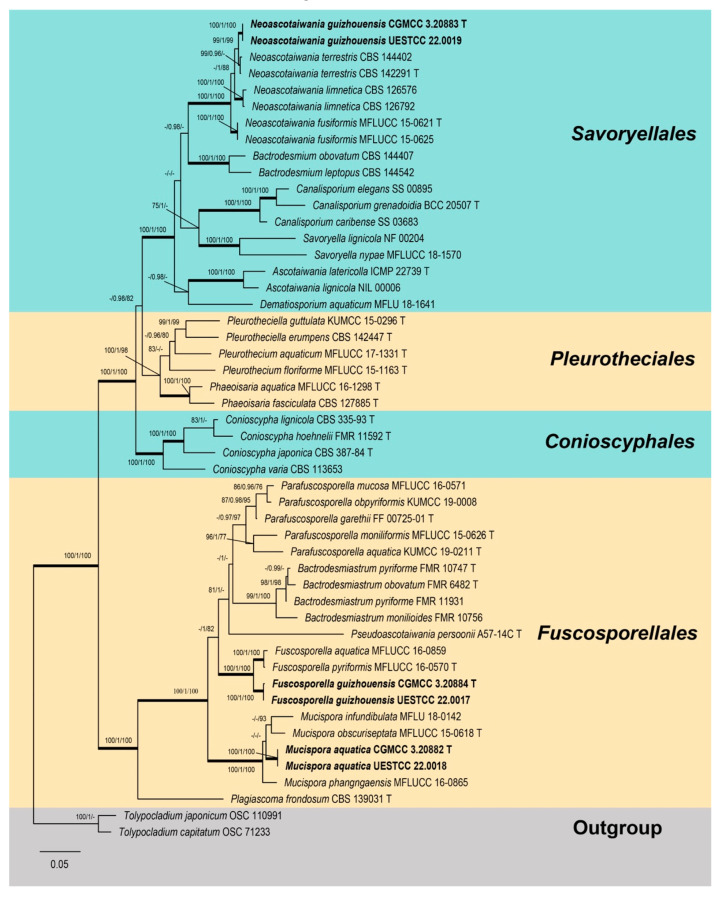
Phylogenetic tree based on the combined LSU, SSU, ITS, *RPB2* and *TEF1α* sequences constructed by maximum likelihood (RAxML) of selected members of Savoryellomycetidae (Sordariomycetes). Thickened branches indicate branch support with MLBS = 100%, PP = 1 and MPBS = 100%. Branch support for ML and MP greater than 75% and BI greater than 0.95 are marked above or below branches as MLBS/PP/MPBS. The abbreviation T indicates the ex-type strain. Species’ names and culture collections in bold are newly collected taxa. The tree was rooted with *Tolypocladium capitatum* (OSC 71233) and *T. japonicum* (OSC 110991).

Five gene loci, LSU, SSU, ITS, *RPB2*, and *TEF1α*, were used to determine the phylogenetic placement of the new collections. The concatenated matrix was comprised of 50 taxa with a total of 4822 characters (LSU: 1–942 bp, SSU: 943–2168 bp, ITS: 2169–2794 bp, *RPB2*: 2795–3871 bp, *TEF1α*: 3872–4822 bp) including gaps. Single-gene analyses were carried out to compare the topologies and clade stabilities, respectively. The results showed that ML, MP and Bayesian inference (BI) were similar in topology without significant conflictions, and these results agree with previous studies [7,16,53]. The best scoring RAxML tree (−ln = −38 991.137) is shown in Figure 1.

In the phylogenetic analyses (Figure 1), isolates of *Fuscosporella guizhouensis* (CGMCC 3.20884 and UESTCC 22.0017) and *Mucispora aquatica* (CGMCC 3.20882 and UESTCC 22.0018) were distributed in Fuscosporellales. Two strains of *Neoascotaiwania guizhouensis* (CGMCC 3.20883 and UESTCC 22.0017) belonged to Savoryellales. *Fuscosporella* *guizhouensis* clustered together with *F. aquatica* (MFLUCC 16-0859) and *F. pyriformis* (MFLUCC 16-0570) and formed a strongly supported monophyletic clade representing the genus *Fuscosporella* (100% MLBS/1.00 PP/100% MPBS). *Mucispora aquatica* nested within the *Mucispora* clade and grouped with *M. infundibulata* (MFLU 18-0142) and *M. obscuriseptata* (MFLUCC 15-0618) without significant support. *Neoascotaiwania guizhouensis* clustered together with *Neoascotaiwania* taxa and was sister to *N. terrestris* (CBS 142,291 and CBS 144402).

## 4. Taxonomy

**Figure 2:**
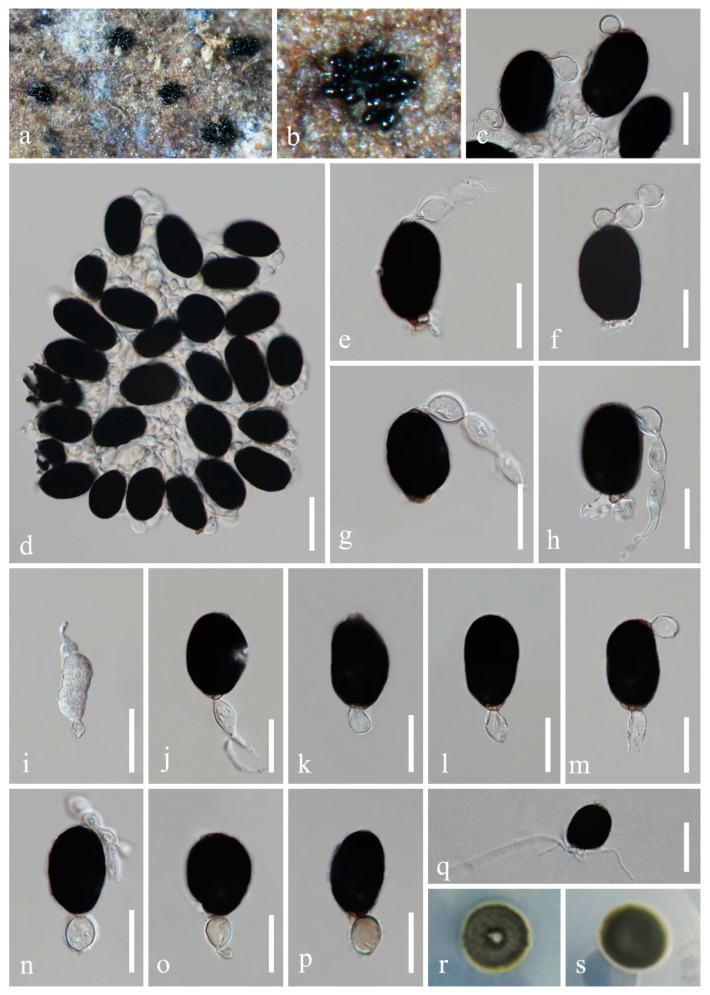
*Fuscosporella guizhouensis* (HKAS 122794, holotype). (**a**,**b**) Colony on submerged wood. (**c**,**d**) Conidiophores with conidia. (**e**–**h**) Conidia with apical appendages. (**i**−**p**) Conidiogenous cells and conidia. (**q**) Germinated conidium. (**r**,**s**) Colony on PDA (r from above, s from below). Scale bars: (**c**) = 30 µm, (**d**) = 40 µm, (**e**−**p**) = 30 µm, (**q**) = 40 µm.

*Fuscosporella guizhouensis* H.Z. Du and Jian K. Liu, sp. nov., Figure 2.

MycoBank number: MB 845466.

Etymology: Referring to the location where the fungus was collected, Guizhou, China.

Holotype: HKAS 122794.

*Saprobic* on decaying wood in freshwater habitat. **Sexual morph:** Undetermined. **Asexual morph:** *Colonies* on natural substrate sporodochial, scattered, black, clustered on substrates. *Mycelium* partly immersed, partly superficial. *Conidiophores* micronematous, indistinct, branched, hyaline, smooth-walled. *Conidiogenous cells* monoblastic, integrated, terminal, globose, subglobose, ellipsoidal or clavate, hyaline to pale brown, 15−26 × 7−15 μm ($\bar{x}$ = 20 × 11 μm, *n* = 20). *Conidia* solitary, acrogenous, ellipsoidal, hyaline when immature, dark brown to black when mature, smooth, (28.5−)42−60 × 24−34 μm ($\bar{x}$ = 49.5 × 29 μm, *n* = 30), with obvious apical appendages, globose to ellipsoidal, or irregular shaped, connected in series.

Culture characteristics: Conidia germinated on WA within 24 h, and germ tubes produced from basal cell. Colonies growing on PDA reached 12−15 mm in diameter after one month at 25 °C, obverse olive to greyish green or dark greyish green in the inner, and light greyish green in the outer ring from above, reverse dark greyish green. *Mycelium* in culture up to 1–3 μm wide, subhyaline to brown, septate, branched. *Conidiophores* and *conidiogenous cells* indistinct. *Chlamydospores* are apparent in culture, globose to ellipsoidal or irregular shaped, hyaline at the beginning, becoming brown to black with ages, 10−21 × 5−19 μm ($\bar{x}$ = 16 × 12 μm, *n* = 30).

Material examined: China, Guizhou Province, Guiyang City, Wudang District, Xiangsihe scenic spot, undisturbed forests with freshwater habitats, 26°26′51″ N, 106°37′53″ E, on decaying wood submerged in a freshwater stream, 22 February 2021, H.Z. Du, S99 (HKAS 122794, holotype); ex-holotype living culture CGMCC 3.20884; *ibid*., HUEST 22.0017, isotype, ex-isotype living culture UESTCC 22.0017.

Notes: *Fuscosporella guizhouensis* resembles *F. pyriformis* in forming sporodochial colonies and dark brown, smooth conidia. However, *F. guizhouensis* has larger conidiogenous cells (15−26 × 7−15 µm vs. 7.5−23 × 3.5−9 μm) and conidia (42−60 × 24−34 µm vs. 23.5−36 × 14−21 μm) [7]. The conidia of *F. guizhouensis* are irregular ellipsoidal, while *F. pyriformis* has obovoid to pyriform conidia. In addition, *F. guizhouensis* is distinguished by the hyaline apical appendages, which are absent in *F. pyriformis* and *F. aquatica* [44]. *Fuscosporella guizhouensis* can be distinguished from *F. aquatica* (17/859 in LSU, 60/524 in ITS) and from *F. pyriformis* (13/814 in LSU, 58/587 in ITS and 52/1024 in *RPB2*). Therefore, *Fuscosporella guizhouensis* is introduced as a new species, and this is the first *Fuscosporella* species reported from China.

**Figure 3:**
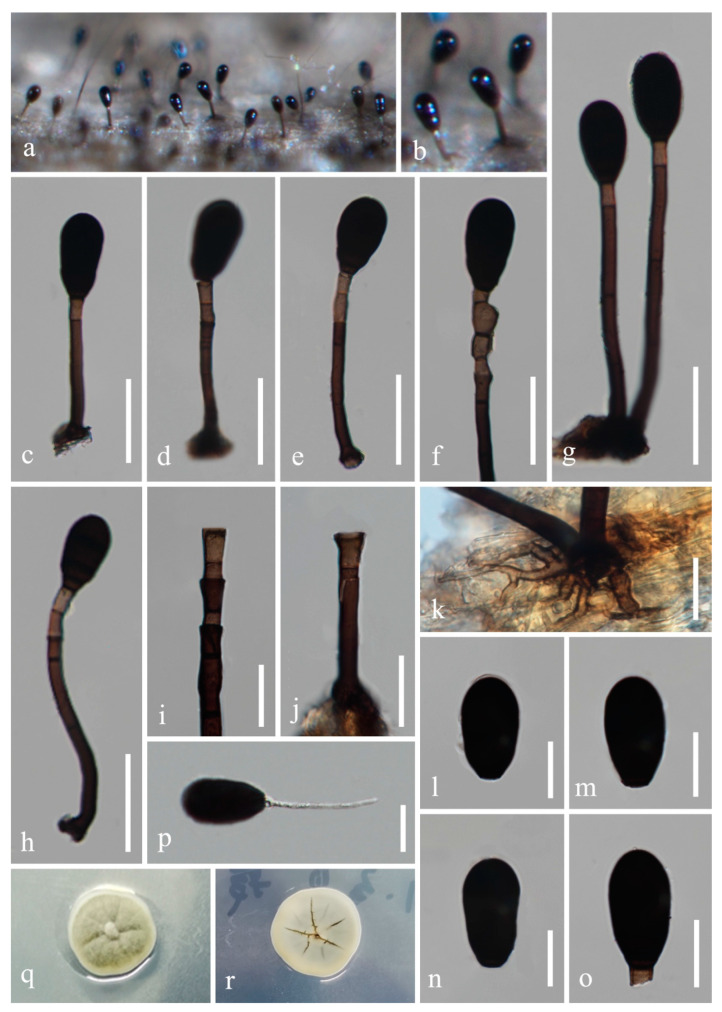
*Mucispora aquatica* (HKAS 122795, holotype). (**a**,**b**) Colonies on submerged wood. (**c**–**h**) Conidiophores with conidia. (**i**,**j**) Conidiogenous cells. (**k**) Mycelium. (**l**–**o**) Conidia. (**p**) Germinated conidium. (**q**,**r**) Colony on PDA ((**q**) from above, (**r**) from below). Scale bars: (**c**−**h**) = 40 µm, (**i**−**p**) = 20 µm.

**Figure 4:**
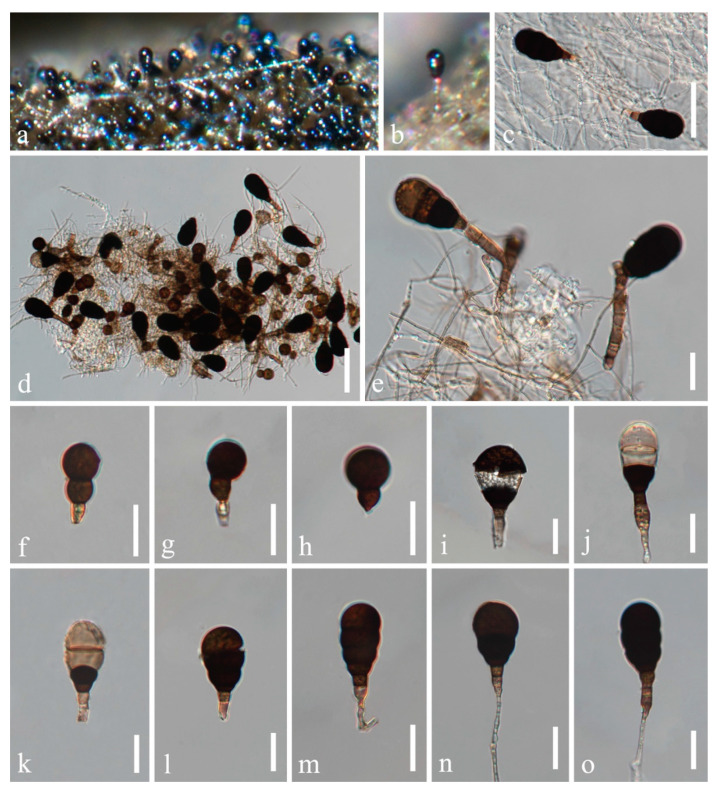
Reproduced asexual morph of *Mucispora aquatica* (CGMCC 3.20882, ex-holotype) on PDA medium. (**a**,**b**) Colonies on PDA. (**c**−**e**) Hyphae and conidiophores with conidia. (**f**−**o**) Conidiogenous cells and conidia. Scale bars: (**c**) = 40 µm, (**d**) = 50 µm, (**e**–**o**) = 20 µm.

*Mucispora aquatica* H.Z. Du and Jian K. Liu, sp. nov., Figure 3 and Figure 4.

MycoBank number: MB 845473.

Etymology: Referring to the aquatic habitat of this fungus.

Holotype: HKAS 122795.

*Saprobic* on decaying wood in freshwater habitat. **Sexual morph:** Undetermined. **Asexual morph:** *Colonies* on natural substrate effuse, glistening, black. *Mycelium* partly immersed, partly superficial, consisting of septate, smooth, hyaline to pale-brown hyphae, (1.5−) 2−4 (−6) um wide. *Conidiophores* macronematous, mononematous, solitary, erect, smooth, mid brown, paler towards the apex, straight or broadly curved, 2−8-septate, (41−)68−128 × 5−7.5 µm ($\bar{x}$ = 104 × 6 µm, *n* = 20), with 1–2 percurrent proliferations. *Conidiogenous cells* monoblastic, integrated, terminal, cylindrical, pale brown to brown, 5−13 × 5−8 µm ($\bar{x}$ = 10 × 6 µm, *n* = 20). *Conidia* acrogenous, ellipsoidal or obovoid, rarely pyriform, rounded at the apex and truncate at the base, smooth, dark brown to black, 34−43 µm ($\bar{x}$ = 37 µm, *n* = 30) long, 17.5−23 µm ($\bar{x}$ = 20 µm, *n* = 30) wide at broadest, 5.5−8 µm ($\bar{x}$ = 7 µm, *n* = 30) wide at the base, septate with dark bands, becoming invisible when mature.

Culture characteristics: Conidia germinated on WA within 24 h, and germ tubes were produced from basal cell. Colonies growing on PDA reached 10−12 mm in diameter after one month at 25 °C, with light greyish green and dense mycelia on the surface, center elevated, reverse light grey. After one month, the diameter did not increase significantly. *Mycelium* subhyaline to pale brown, 2.5−4 µm wide in culture. *Conidiophores* light brown to brown, 14.5−37 × 4−6 µm ($\bar{x}$ = 24 × 5 µm, *n* = 20). *Conidiogenous cells* integrated, subhyaline to pale brown, 5−7.5 × 5−8 µm ($\bar{x}$ = 6 × 7 µm, *n* = 20). *Conidia* pale brown to black, 1−4-septate, mostly 2-septate, globose to obovoid, rounded at the apex and truncate at the base, smooth, constricted at the septa, 33−39 µm ($\bar{x}$ = 36 µm, *n* = 30) long × 19−23 µm ($\bar{x}$ = 21 µm, *n* = 30) wide at broadest, 5.5−9 um ($\bar{x}$ = 7 µm, *n* = 30) wide at base.

Material examined: CHINA, Guizhou Province, Guiyang City, Wudang District, Xiangsihe scenic spot, undisturbed forests with freshwater habitats, 26°26′51″ N, 106°37′53″ E, on decaying wood submerged in a freshwater stream, 22 February 2021, H.Z. Du, S95 (HKAS 122795, holotype); ex-holotype living culture CGMCC 3.20882; *ibid*., HUEST 22.0018, isotype, ex-isotype living culture UESTCC 22.0018.

Notes: *Mucispora aquatica* resembles *M. obscuriseptata*, *M. phangngaensis* and *M. infundibulate* in forming scattered, dark brown to black colonies, macronematous, mononematous, solitary, erect, smooth conidiophores and acrogenous, ellipsoidal to obovoid conidia. However, *M. aquatica* is distinguished from *M. obscuriseptata* by the absence of conidial sheath [7]. The conidiophores of *M. aquatica* (68−128 µm) are smaller than those of *M. obscuriseptata* (80−170 µm) and *M. phangngaensis* (170−305 µm) [44] but larger than *M. infundibulata* (50−60 µm). *Mucispora infundibulata* is unique in its inflated cupulate conidiogenous cells [11]. In addition, *Mucispora aquatica* can be distinguished from *M*. *infundibulata* (30/836 in LSU, 70/606 in ITS); from *M. phangngaensis* (27/844 in LSU, 58/575 in ITS); and from *M. obscuriseptata* (35/861 in LSU, 69/605 in ITS and 60/879 in RPB2). Phylogenetic analysis (Figure 1) showed that *Mucispora aquatica* has a close phylogenetic relationship with *M*. *infundibulata* and *M. obscuriseptata*, but it can be recognized as a distinctly phylogenetic species. Therefore, we introduced *Mucispora aquatica* as a new species based on morphology and phylogeny.

**Figure 5:**
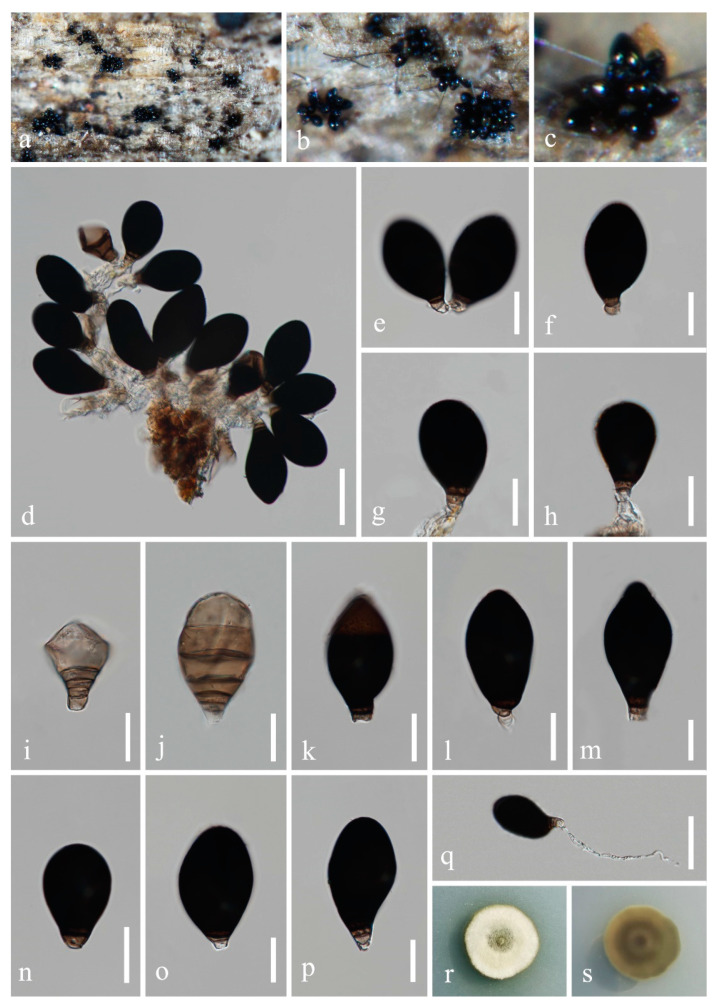
*Neoascotaiwania guizhouensis* (HKAS 122796, holotype). (**a**−**c**) Colonies on submerged wood. (**d**−**h**) Conidiophores with conidia. (**i**−**p**) Conidia. (**q**) Germinated conidium. (**r**,**s**) Colony on PDA ((**r**) from above, **s** from below). Scale bars: (**d**) = 40 µm, (**e**−**p**) = 20 µm, (**q**) = 40 µm.

*Neoascotaiwania guizhouensis* H.Z. Du and Jian K. Liu, sp. nov., Figure 5.

MycoBank number: MB 845474.

Etymology: Referring to the location where the fungus was collected, Guizhou Province, China.

Holotype: HKAS 122796.

*Saprobic* on decaying wood in freshwater habitat. **Sexual morph:** Undetermined. **Asexual morph:** *Colonies* on natural substrate sporodochial, glistening, black, clustered on substrates. *Mycelium* partly immersed, partly superficial. *Conidiophores* micronematous, mononematous, hyaline to pale brown, smooth, thin-walled. *Conidiogenous cells* monoblastic, cylindrical, hyaline to pale brown. *Conidia* solitary, ellipsoidal, pyriform to obovoid, broadly rounded or cuneate at the apex, 3−6 septate, pale brown when young, becoming dark brown to black when mature, paler at the basal cell, 49−62(−68) × 29−36(−39) μm ($\bar{x}$ = 56 × 32 μm, *n* = 30).

Culture characteristics: Conidia germinated on WA within 24 h, and germ tubes produced from basal cell. Colonies growing on PDA reached 8−10 mm in diameter after one month at 25 °C, with white and dense *mycelium* on the surface, the center greyish green, reverse greyish brown and with a dark greyish brown ring in the middle. After one month, the diameter did not increase significantly. *Mycelium* hyaline to brown, septate, branched, 2−4 μm ($\bar{x}$ = 3 μm, *n* = 30) wide, *Chlamydospores* are apparent, hyaline at the beginning, becoming brown or dark brown, 8−13 × 6−10 ($\bar{x}$ = 11 × 9 μm, *n* = 30).

Material examined: CHINA, Guizhou Province, Guiyang City, Wudang District, Xiangsihe scenic spot, undisturbed forests with freshwater habitats, 26°26′51″ N, 106°37′53″ E, on decaying wood submerged in a freshwater stream, 22 February 2021, H.Z. Du, S95-2 (HKAS 122796, holotype); ex-holotype living culture CGMCC 3.20883; *ibid*., HUEST 22.0019, isotype, ex-isotype living culture UESTCC 22.0019.

Notes: *Neoascotaiwania guizhouensis* resembles *N. limnetica* and *N. terrestris* in forming dark, effuse colonies consisting of single, dark brown, transversely septate conidia. However, *N. guizhouensis* has larger conidia (49–68 × 29–39 μm) than those of *N. limnetica* (23–39 × 14.5–18.5 μm) and *N. terrestris* (25.5−44.5 × 13–22 μm) [8,14,16]. Furthermore, *N. guizhouensis* differs from *N. fusiformis* by its micronematous conidiophores, while the latter has macronematous conidiophores [7]. Additionally, *Neoascotaiwania guizhouensis* can be distinguished from *N. terrestris* (35/1017 in SSU, 14/554 in ITS, 13/1069 in *RPB2* and 18/938 in *TEF1α*); from *N. limnetica* (13/862 in LSU, 36/561 in ITS, 36/845 in *RPB2* and 28/884 in *TEF1α*); and from *N. fusiformis* (16/860 in LSU, 34/594 in ITS and 35/817 in *RPB2*). In our phylogenetic tree (Figure 1), *Neoascotaiwania guizhouensis* was sister to *N. terrestris*, but they are distinguishable in morphology and phylogeny. Therefore, we introduced *Neoascotaiwania guizhouensis* as a new species.

## 5. Discussion

The phylogenetic analyses based on the combined gene regions (LSU, SSU, ITS, *RPB2* and *TEF1α*) placed three new species, *Fuscosporella guizhouensis*, *Mucispora aquatica* and *Neoascotaiwania guizhouensis*, in Fuscosporellaceae and Savoryellaceae (Savoryellomycetidae, Sordariomycetes) and are described in asexual stages without known sexual morphs. Species in *Fuscosporella* and *Mucispora* are reported from freshwater habitats in Thailand and China [7,11,44,54]; they may be exclusive in freshwater habitats. In this study, we provide the first record of *Fuscosporella* in China. *Neoascotaiwania* taxa are widely distributed in France, Spain and Thailand [7,8,14]. *Neoascotaiwania guizhouensis*, *N. fusiformis*, and *N. limnetica* are also found on decaying submerged wood in freshwater habitats [7,8,14,16,55], while *N. terrestris* was isolated from soil [14], which indicates that they are widely distributed and not limited by the growth environment.

The sexual morph of *Neoascotaiwania* differs from *Ascotaiwania* in having cylindrical asci with a thinner, non-amyloid and discoid apical ring, different septate ascospores and bactrodesmium-like asexual morph [14]. Besides, *Ascotaiwania* has monodictys-like [56], monotosporella-like [57,58] and trichocladium-like [56] asexual morphs. Dayarathne et al. [59] synonymized *Neoascotaiwania* under *Ascotaiwania* based on similar morphology and multi-gene phylogeny analysis. However, recent studies showed that *Neoascotaiwania* and *Ascotaiwania* were not congeneric [16,60]. We follow this treatment and treat *Ascotaiwania* and *Neoascotaiwania* as distinct genera.

Multi-locus phylogenetic analysis has been crucial for delimiting the novel fungi [61]. The use of multi-gene datasets to infer phylogenetic relationships has dramatically improved the resolution, especially when protein genes are combined with other genes, and the solution substantially increased [62,63]. For *Fuscosporella* and *Mucispora*, ITS, LSU and SSU rDNA datasets are available for all the species [7,11,44,54]. However, for the protein genes, only two species had the *RPB2* sequence (unverified), and no *TEF1α* dataset. Therefore, the problem of low similarity occurred after the blastn search without a corresponding sequence in the same genus for alignment. This study provides the *RPB2* and *TEF1α* sequences of *Fuscosporella* and *Mucispora*, which make up for the lack of protein genes in these two genera.

## Data Availability

The sequences data were submitted to GenBank.
